# A salen-based dinuclear cobalt(ii) polymer with direct and indirect synergy for electrocatalytic hydrogen evolution†

**DOI:** 10.1039/d5sc02073e

**Published:** 2025-05-02

**Authors:** Xiao-Mei Hu, Wen-Jie Shi, Jian-Hua Mei, Yu-Chen Wang, Wei-Xue Tao, Di-Chang Zhong, Tong-Bu Lu

**Affiliations:** a Institute for New Energy Materials and Low Carbon Technologies, School of Materials Science and Engineering, Tianjin University of Technology Tianjin 300384 China lutongbu@tjut.edu.cn dczhong@email.tjut.edu.cn wjshi@email.tjut.edu.cn

## Abstract

Optimizing the spatial arrangement and geometric configuration of dinuclear metal sites within catalysts to leverage the dinuclear metal synergistic catalysis (DMSC) effect is a promising strategy for enhancing catalytic performance. In this work, we report a salen-based dinuclear cobalt covalent organic polymer (Co_2_-COP) that exhibits both direct and indirect DMSC synergistic effects, significantly improving catalytic efficiency for the electrocatalytic alkaline hydrogen evolution reaction (HER). Notably, one of the Co atoms in this structural unit features an OH^−^ anion. The OH^−^ anion facilitates both H_2_O adsorption through p–p orbital overlapping interaction and the subsequent OH* intermediate removal by pre-attracting cations. As a result, Co_2_-COP exhibits superior HER activity that surpasses its single-atom counterpart by a factor of 36. Control experiments and theoretical calculations revealed that the enhanced catalytic efficiency of Co_2_-COP is attributed to both the direct DMSC effect between two Co^II^ ions, and the indirect DMSC involving the OH^−^ anion and alkali cations. This synergistic interaction significantly facilitates water activation and accelerates the removal of the OH* intermediate, all of which are intricately linked to the unique dinuclear structure of the material.

## Abstract 2

A salen-based dinuclear cobalt polymer exhibits both direct and indirect DMSC effects, where the Co-OH unit promotes H_2_O activation and OH removal, enabling 36-fold higher activity than the single-atom counterpart *via* synergistic.

## Graphical abstract

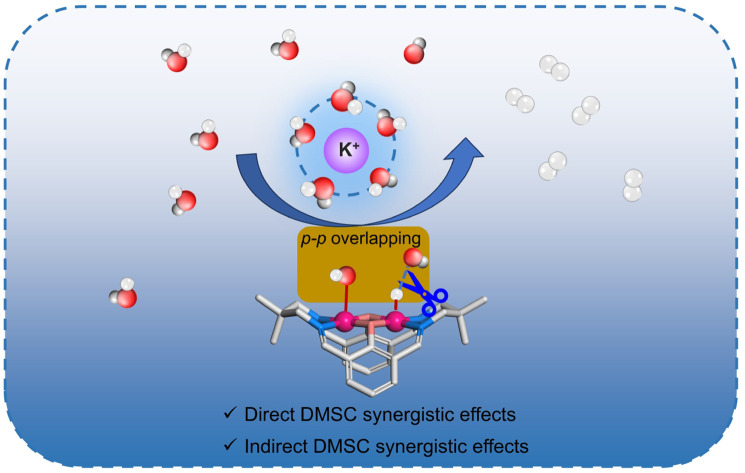

## Introduction

The dinuclear metal synergistic catalysis (DMSC) effect has emerged as a promising strategy for enhancing catalytic performance in energy-related applications.^1–5^ This effect is facilitated by the presence of two metal centers within the catalyst, which work synergistically to boost reactivity and selectivity.^6^ In dinuclear catalysis, two distinct synergistic effects are typically observed: direct and indirect synergistic effects. The direct synergistic effect arises from the interaction between the two metal centers, typically involving electronic transfer,^7^ metal–metal interactions^8^ or cooperative coordination,^9^ which can accelerate substrate activation and facilitate reaction steps. On the other hand, the indirect synergistic effect is mediated by the ligands^10^ or solvent molecules^11^ in the reaction environment, which help the active center to stabilize key reaction intermediates or promote efficient charge transfer. While the majority of studies have focused on these effects in isolation,^12,13^ their coexistence and interplay within a single catalytic system remain largely underexplored.

**Scheme 1:**
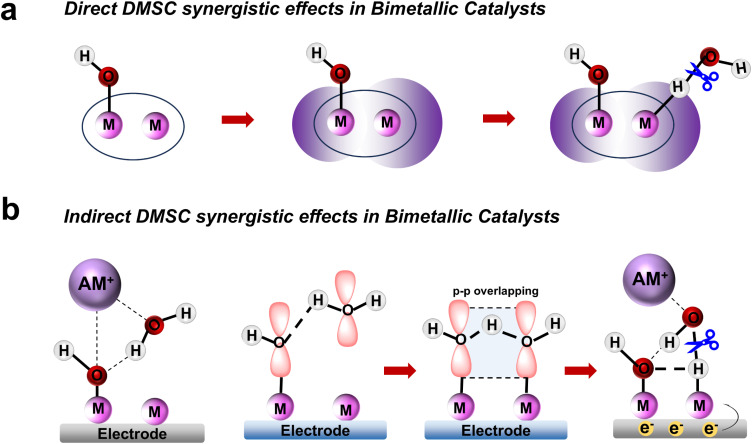
(a) Schematic diagram illustrating the direct DMSC effects in dinuclear catalysts to promote H_2_O dissociation. (b) Schematic diagram depicting the indirect DMSC effects to enhance H_2_O dissociation.

In electrocatalytic hydrogen evolution reactions (HER),^14–18^ dinuclear metal complexes enhance catalytic performance through both direct metal–metal interactions that facilitate proton reduction and indirect contributions from the ligands that stabilize water and modulate the activation of reactants. Salen-based dinuclear metal coordination complexes are gaining recognition as promising candidates for advanced catalytic systems due to their ability to tune the electronic properties of metal centers and enable cooperative catalysis (Scheme 1a). Moreover, these salen-based complexes have the potential to adsorb OH^−^ anion at one metal site. This OH^−^ anion can form hydrogen bond with H_2_O, strengthening the adsorption and stabilization of H_2_O adsorbed at the other metal site through spatial interaction involving the p orbitals of the O atoms (Scheme 1b).^19^ Furthermore, the adsorbed OH^−^ anions attract alkali metal cation hydrates *via* electrostatic attraction, thereby facilitating the removal of the OH* intermediate and promoting the continuous progression of the reaction.

Dinuclear cobalt systems represent a key class of DMSC-based catalysts, offering a well-balanced platform that combines electronic flexibility, redox tunability, and cooperative interactions.^20–22^ Recent studies have focused on engineering ligand environments^23^ adjusting metal–metal coordination geometry,^24^ and incorporating secondary interactions^11^ to enhance both catalytic activity and selectivity. Nevertheless, a systematic understanding of how direct and indirect synergistic effects interplay to enhance catalytic performance in dinuclear cobalt systems remains limited.

**Fig. 1:**
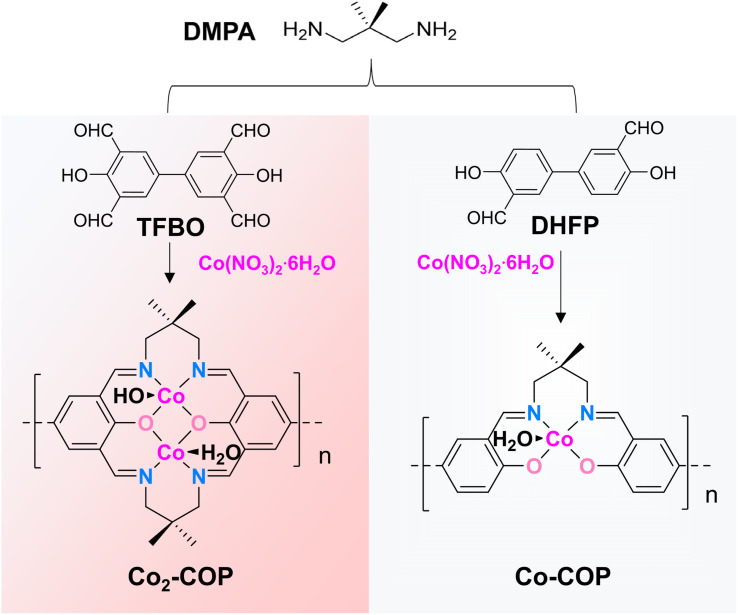
Schematic illustration of the synthetic process and structure of Co_2_-COP and Co-COP.

Herein, we report a dinuclear cobalt covalent organic polymer (Co_2_-COP) with well-defined Co-Robson units as an efficient electrocatalyst for alkaline HER (Fig. 1). Co_2_-COP exhibits significantly higher activity than its single-atom counterpart. Mechanistic studies reveal that adjacent Co atoms modulate the electronic environment to optimize intermediate adsorption (direct synergy), while surface-adsorbed OH^−^ and hydrated K^+^ promote OH* desorption (indirect synergy). This work offers new insights into dual synergistic mechanisms in dinuclear cobalt catalysis under alkaline conditions.

## Results and discussion

### Preparation and characterization

**Fig. 2:**
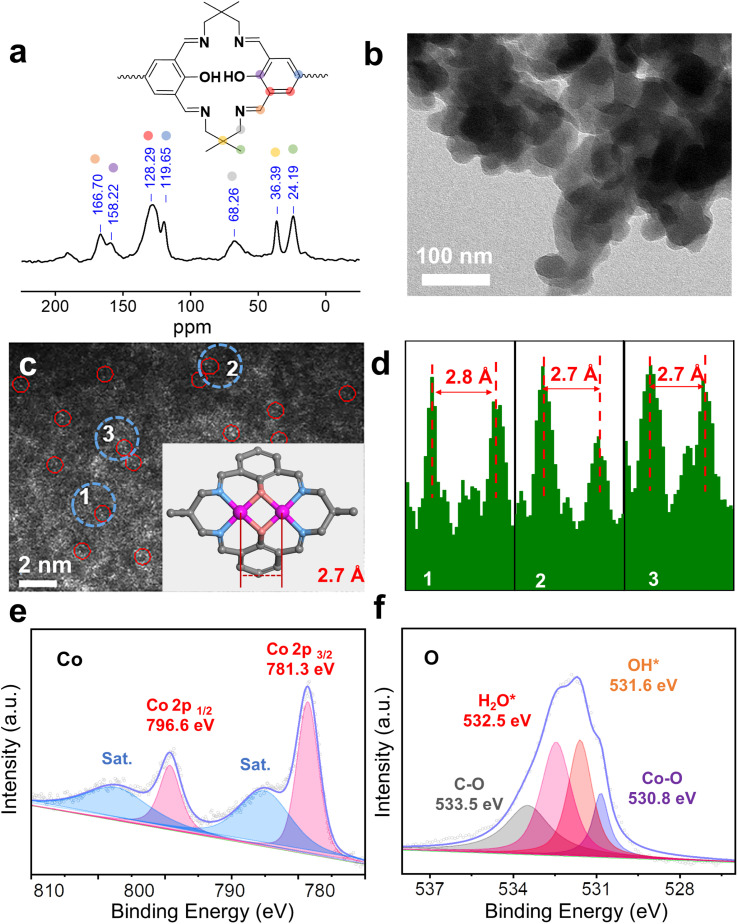
(a) ^13^C solid-state NMR spectra of TFBO-COP. (b) TEM image of Co_2_-COP. (c) HAADF-STEM image and the dinuclear cobalt distance in Co_2_-COP. (d) Intensity profiles corresponding to different areas of dinuclear cobalt in Co_2_-COP. XPS spectra of Co 2p (e) and O 1s (f) for Co_2_-COP.

The framework of Co_2_-COP, designated as TFBO-COP, was synthesized by condensing 2,2-dimethyl-1,3-propanediamine (DMPA) with 3,3′,5,5′-tetraformyl-4,4′-biphenyldiol (TFBO) (Fig. S1 and S2†). And Co_2_-COP was subsequently obtained by metalating TFBO-COP with Co(NO_3_)_2_·6H_2_O (Fig. 1 and S4†).^25^ Similarly, Co-COP containing mononuclear Co(ii)-salen units was synthesized by metalating DHFP-COP, which was prepared by replacing TFBO with 4,4′-dihydroxy-3,3′-diformylbiphenyl (DHFP) in the condensation process (Fig. 1, S3 and S4†). The powder X-ray diffraction (XRD) found that both Co_2_-COP and Co-COP exhibit amorphous characteristics (Fig. S5†). The Fourier-transform infrared (FT-IR) spectra of TFBO and DHFP exhibit prominent C=O stretching vibration at 1672 and 1660 cm^−1^, respectively. These bands disappear in Co_2_-COP and Co-COP spectra, where a new absorption bands at 1635 cm^−1^ appears, which can be attributed to the stretching vibration of C=N bond (Fig. S6†).^26^ This shift indicates the successful synthesis of Co_2_-COP and Co-COP *via* Schiff-base condensation. In the solid-state ^13^C cross-polarized magic angle spinning (CP-MAS) NMR spectrum of TFBO-COP, a distinct signal at 166.7 ppm corresponds to the C=N group,^27^ along with signals from aromatic and alkyl carbons (Fig. 2a and S7†), further supporting the formation of these materials through Schiff-base chemistry. Scanning electron microscopy (SEM) (Fig. S8 and S9†) and transmission electron microscopy (TEM) images (Fig. 2b) reveal block-like morphologies for both Co_2_-COP and Co-COP. Energy-dispersive X-ray spectroscopy (EDX) mapping confirms the homogeneous distribution of Co, N and O elements throughout both materials (Fig. S8 and S9†). The aberration-corrected HAADF-STEM image of Co_2_-COP shows paired bright dots (Fig. 2c), indicative of diatomic Co. The distance between two Co pair is approximately 2.7 Å, which matches the simulated Co–Co distance (2.7 Å) in Co_2_-COP (Fig. 2d). In contrast, Co atoms in Co-COP are separated by about 1.1 nm, in agreement with the simulated distance (Fig. S10†).

X-ray photoelectron spectroscopy (XPS) analysis confirmed the presence of C, N, O and Co in both polymers (Fig. S11a and S12a†). Co(2p) spectra show peaks at 781.3 and 796.6 eV for Co_2_-COP, and 781.2 and 796.3 eV for Co-COP, corresponding to Co 2p_3/2_ and Co 2p_1/2_, respectively (Fig. 2e and S12b†). These results indicate a +2 oxidation state for Co species in both materials.^28^ The N 1s spectra of both Co_2_-COP and Co-COP exhibit peaks at 399.2 and 399.3 eV, associated with Co–N coordination (Fig. S11b and S12d†).^29,30^ The O 1s spectra of Co_2_-COP show peaks at 530.8, 531.6, 532.5 and 533.5 eV, assigned to Co–O, OH*, H_2_O* and C–O,^26^ with a near 1 : 1 ratio of OH* to H_2_O* (Fig. 2f).^31–33^ In contrast, the O 1s spectra of Co-COP displays three peaks at 531.0, 532.0, and 533.1 eV, attributed to Co–O, H_2_O*, and C–O, (Fig. S12c†) indicating the absence of OH* in Co-COP. Inductively coupled plasma optical mass spectrometry (ICP-MS) showed that Co_2_-COP contains 11.80 ± 0.40 wt% of Co, while Co-COP contains 10.43 ± 0.30 wt%, slightly lower than the theoretical values of 14.80% and 11.99%, respectively (Table S1†).

### Electrocatalytic HER

**Fig. 3:**
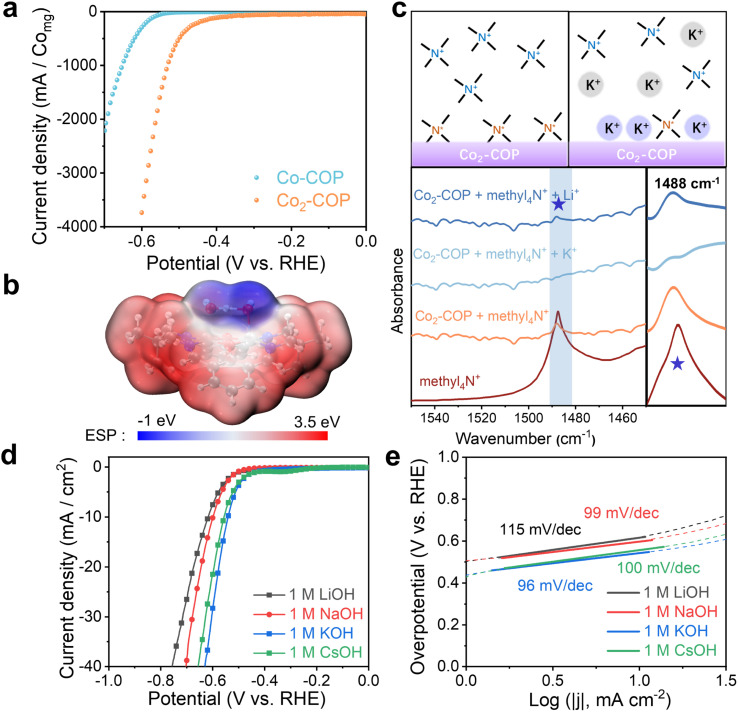
(a) HER polarization curves of Co_2_-COP and Co-COP in 1 M KOH aqueous solution. (b) Visualization of surface electrostatic potentials of Co_2_-COP. (c) Schematic diagram of FT-IR spectral interpretation. In the absence of AM^+^, the methyl_4_N^+^ adsorbed on the surface of Co_2_-COP as evidenced by increase in the ∼1488 cm^−1^ band, in the presence of K^+^ and Li^+^, methyl_4_N* is replaced by K^+^ or Li^+^ as indicated by weaken the ∼1488 cm^−1^ band in the infrared spectrum. HER polarization curves (d) and Tafel plots (e) of Co_2_-COP in 1.0 M AMOH (AM^+^ = Li^+^, Na^+^, K^+^ and Cs^+^).

The HER activity of Co_2_-COP and Co-COP were evaluated through linear sweep voltammetry (LSV) in a three-electrode setup using an N_2_-saturated 1 M KOH solution. Co_2_-COP demonstrated a catalytic reduction current density of 10 mA cm^−2^ at an overpotential (*η*) of 540 mV, which is significantly lower than that of Co-COP (*η* = 669 mV) (Table S2†). This overpotential corresponds to a specific current of 1233.7 mA mg_Co_^−1^ for Co_2_-COP (Fig. 3a), which is 36 times greater than that of Co-COP (34 mA mg_Co_^−1^). The Tafel slope of Co_2_-COP is 96 mV dec^−1^, notably lower than Co-COP 135 mV dec^−1^ (Fig. 3e and S23b†), further indicating its superior electrocatalytic performance. The Tafel slope analysis suggests that the HER mechanism for Co_2_-COP follows the Volmer–Heyrovsky pathway, whereas Co-COP predominantly follows the Volmer step.

To further understand the superior HER activity of Co_2_-COP, electrochemical impedance spectroscopy (EIS) were conducted. The results show that Co_2_-COP exhibits a charge transfer resistance (*R*_ct_) of 89.38, which is significantly lower than that of Co-COP (*R*_ct_ = 344.5), indicating faster charge transfer kinetics for Co_2_-COP (Fig. S13 and Table S3†). Additionally, the electrochemically active surface area (ECSA) was estimated by measuring the double-layer capacitance (*C*_dl_) through cyclic voltammetry (CV) in the non-faradaic region (0–0.2 V *vs.* RHE). Co_2_-COP shows a *C*_dl_ value of 0.47 mF cm^−2^, similar to that of Co-COP (0.53 mF cm^−2^; Fig. S14 and Table S2†), suggesting that both materials offer similar accessible surface areas for the HER reaction. This implies that the enhanced HER performance of Co_2_-COP is due to the synergistic catalytic effect of the diatomic Co centers.

Further electrochemical assessments revealed that Co_2_-COP exhibits excellent electrocatalytic HER performance over a wide electrochemical window. As shown in Fig. S15a,† Co_2_-COP demonstrates an averaged faradaic efficiency (FE) of 86.7% in the overpotential range of from 500 to 700 mV, with the turnover frequency (TOF) increasing almost parabolically with *η* value. At *η* = 700 mV, the H_2_ production rate reaches 12.0 s^−1^, which is double that of Co-COP (Fig. S16†).^34^ Co_2_-COP also exhibits good electrochemical stability, as confirmed by long-term cyclic voltammetry and time-dependent potential measurements. After 3000 cycles, the polarization curves of Co_2_-COP displayed no significant potential decay (Fig. S15b†), and it maintains stable performance during 24 hours of continuous electrolysis at electrolysis at 10 mA cm^−2^ (inset Fig. S15b†). After the stability measurements, Co_2_-COP retained the block morphology (Fig. S17†), and no discernible change was observed in the FT-IR (Fig. S18†). The Co species remained in the +2 oxidation state (Fig. S19†). ICP-MS analyses demonstrated negligible leaching of Co (<0.4%) after electrolysis. These observations validate the exceptional stability of Co_2_-COP.

To identify the active sites in Co_2_-COP, we performed control experiments using metal-free TFBO-COP, DHFP-COP, and bare GC instead of Co_2_-COP. The current response of these materials is particularly low, indicating a lack of HER activity (Fig. S20†). This result indicates that Co(ii) serves as the catalytic active site in Co_2_-COP. The differential pulse voltammogram of Co_2_-COP represented a Co^II^–Co^I^ redox peak at −0.54 V, which closely resembles that of Co-COP (−0.57 V) (Fig. S21†).^35^ When 1 mL of H_2_O was added as the proton source, the Co^I/II^ anodic peak vanished and the current density attributed to catalytic proton reduction increased (Fig. S22†). This observation suggests that the electrocatalytic HER mechanism is similar in Co_2_-COP and Co-COP, most likely *via* the Heyrovsky pathway, involving a Co^I^–H intermediate.^36^

Given the superior HER activity of Co_2_-COP, we hypothesis that the presence of AM^+^ (AM^+^: alkali metal cations) may play a crucial role in this enhancement. As Fig. 3b shown, the electrostatic potential (ESP) map reveals a blue region surrounding the OH group in Co_2_-COP, favoring the adsorption of positive cations from the solution. This leads to the hypothesis that AM^+^ cations can bind to the Co_2_-COP surface. To verify this hypothesis, we utilized tetramethylammonium (methl_4_N^+^) as the vibrational probe to detect AM^+^ cations adsorption.^37,38^ As shown in Fig. 3c, the absorbed methyl_4_N in Co_2_-COP presented the characteristic peak at ∼1488 cm^−1^. After treatment with a 100 mM KOH solution, this peak disappeared, whereas treatment with a 100 mM LiOH solution caused only a slight decrease in peak intensity. These results confirm that AM^+^ cations bind to the Co_2_-COP surface, with K^+^ showing higher adsorption capacity than Li^+^.

We then investigated the HER activity of Co_2_-COP in alkaline media containing different alkali metal cations (AMOH solution; AM^+^ = Li^+^, Na^+^, K^+^, and Cs^+^). As expected, Co_2_-COP exhibited the highest HER activity in KOH, with an activity order of K^+^ > Cs^+^ > Na^+^ > Li^+^ (Fig. 3d). This was in contrast to Co-COP, while the HER activity followed the order Cs^+^ < K^+^ < Na^+^ < Li^+^ (Fig. S23†). The Tafel slope for Co_2_-COP in different AMOH solutions was consistently below 120 mV dec^−1^, indicating a Volmer–Heyrovsky mechanism. Notably, the Tafel slope values correlated with the HER activity order, with the lowest value (and highest activity) observed in KOH.

To examine the influence of cation concentration on HER kinetics (Table S4†). The HER activity of Co_2_-COP and Co-COP increased significantly with higher concentrations of K^+^, Na^+^, and Li^+^ (Fig. S24 and S25†), and the reaction order with respect to cation concentration were positive. Specifically, the HER reaction order for K^+^ was higher in Co_2_-COP (around 0.8) compared to Na^+^ (around 0.6) and Li^+^ (around 0.5) (Fig. S26†). In contrast, the ranked list in Co-COP is Li^+^ > Na^+^ > K^+^, and their HER reaction orders were approximately 1.3, 0.7, and 0.6, respectively (Fig. S27†). These sequences are also consistent with their activity trends. These findings also suggest that increased cation concentration near the surface of Co_2_-COP enhances HER kinetics, with K^+^ being the most effective species for promoting adsorption and modifying the rate-determining step (RDS) of the HER process.

### Direct and indirect DMSC effect investigation

**Fig. 4:**
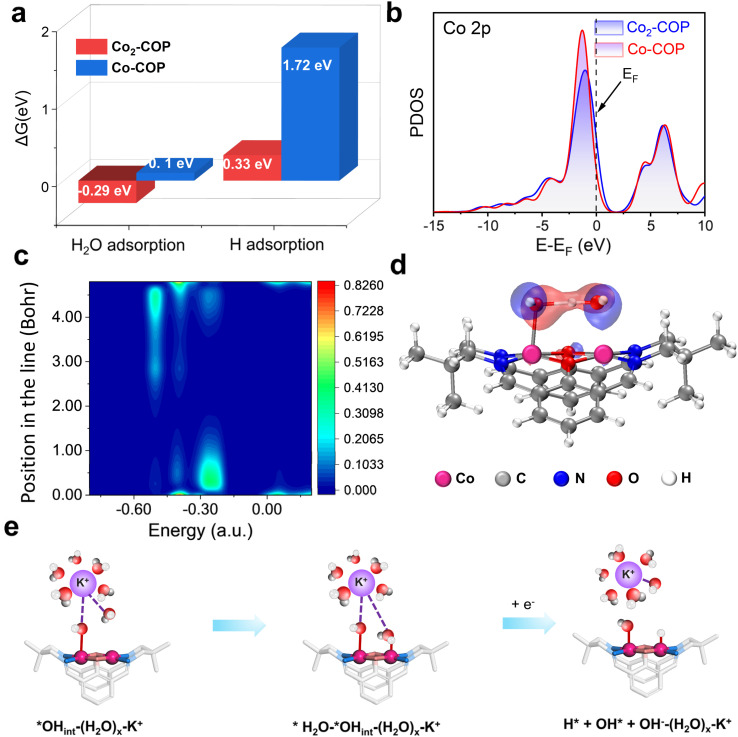
(a) The calculated water adsorption and hydrogen adsorption free-energy diagram (Δ*G*_H_2_O*_) of Co_2_-COP (red) and Co-COP (blue). (b) PDOS consisting of Co_2_-COP and Co-COP. The Fermi level is set to 0 eV. LDOS (c) and the orbital interactions (d) between the O atom of the OH^−^ anion and water in Co_2_-COP, with water adsorbed on the Co site. (e) Mechanism diagram of (H_2_O)_*x*_–K^+^ synergically promoting alkaline HER for Co_2_-COP.

Understanding both the direct and indirect DMSC effect of diatomic catalysts on HER is crucial for the design of high-performance electrocatalysts. To explore the direct interaction of diatomic Co in Co_2_-COP, we performed density functional theory (DFT) calculations. In alkaline media, the HER follows a two-electron transfer process, involving the Volmer, Heyrovsky, and Tafel steps. Water dissociation which is initiated in the Volmer step is crucial for H* generation. Electrochemical HER tests indicate that water dissociation kinetics play a vital role in the activity of both Co_2_-COP and Co-COP. Hence, we simulated the adsorption of both H_2_O and H species on the individual structures (Fig. S28†). The energy profile for water adsorption reveals that Co_2_-COP has a lower energy barrier of −0.29 eV compared to Co-COP's 0.1 eV, indicating that the diatomic Co in Co_2_-COP significantly enhances water adsorption and dissociation kinetics. Moreover, Co_2_-COP shows more favorable hydrogen adsorption with a Δ*G*_H_ value of 0.33 eV, compared to Co-COP's 1.72 eV (Fig. 4a). A comparison of the projected density of states (PDOS, Fig. 4b) further highlights how the adjacent Co atoms in Co_2_-COP effectively enhances the total d-electron domination of the catalyst near the Fermi level, which will benefit H_2_O activation and lead to energetically catalytic activity.^39^ This suggests that the diatomic Co centers in Co_2_-COP interact synergistically, optimizing reactant and product adsorption to promote efficient HER.

Moreover, the adjacent Co atom's OH group in Co_2_-COP forms a hydrogen bond with H_2_O, with a bond length of 1.50 Å (Fig. S28†). Notably, the local density of states (LDOS) and orbital spectra of Co_2_-COP reveal that the adsorbed H_2_O is stabilized by the OH^−^ anion, which interacts with the H_2_O at the adjacent Co site through spatial interactions involving the p orbitals of the oxygen atoms (Fig. 4c and d). These results showing the indirect synergistic effect of the diatomic catalyst promotes H_2_O dissociation. Subsequently, we investigated the indirect influence of cations on HER kinetics in Co_2_-COP. To understand the pH effect on HER kinetics, we adjusted the pH of the electrolyte and observed the response for both Co_2_-COP and Co-COP (Table S5†). As shown in Fig. S29a,† no pH dependence for the kinetics of HER in Co_2_-COP under alkaline conditions on the NHE scale, whereas Co-COP exhibits pH dependence (Fig. S29d†), likely due to differences in the RDS between the two catalysts. The Tafel slope for Co_2_-COP remained below 120 mV dec^−1^, consistent with a Volmer–Heyrovsky mechanism (Fig. S29b and e†). Conversely, the RDS of HER by Co-COP remained the Volmer step at all times.^40^ These observations suggest that the cation preconcentration on the surface of Co_2_-COP had positively impacts the HER kinetics on the RHE scale. Remarkably, the reaction order of HER in Co_2_-COP varies with cation concentration, as demonstrated in Fig. S29c and f,† where the reaction order decrease from 0.66 to 0.24 as the voltage shifts from −0.6 to −0.72 V, in contrast to the constant order observed for Co-COP.

Herein we proposed the indirect DMSC effect mechanism for Co_2_-COP based on the hard–soft acid–base (HSAB) theory established in the literature.^41^ In this framework, K^+^ is classified as a Lewis acid, and the OH groups (*OH_Co_) in the Co_2_-COP catalyst function as a Lewis base. As a result, the (H_2_O)_*x*_–K^+^ adduct in the electrical double layer region is attracted to the surface of Co_2_-COP. The full Volmer mechanism involves the adsorption and dissociation of water molecules. Therefore, the step of Co_2_-COP involving (H_2_O)_*x*_–K^+^ can be divided into two separate steps (Fig. 4e):

2a H_2_O* + *OH_Co_–(H_2_O)_*x*_–K^+^ → H* + *OH_Co_ + OH^−^–(H_2_O)_*x*_K^+^

2b H* + *OH_Co_ + OH^−^–(H_2_O)_*x*_–K^+^ → H* + *OH_Co_ + H_2_O + OH^−^–(H_2_O)_*x*_–K^+^

The affinity of hard Lewis acid K^+^ towards hard Lewis base OH^−^ is strong, thus promoting OH^−^ desorbing into the bulk and encouraging the Volmer step in a unidirectional manner,^42,43^ the diatomic Co in Co_2_-COP worked with cations finished the indirect synergistic catalysis.

The driving force for the OH^−^ desorption weakens in the order of Li^+^ > Na^+^ > K^+^ > Cs^+^, but the sequential trend for HER activity of Co_2_-COP is K^+^ > Cs^+^ > Na^+^ > Li^+^. This apparent anomaly may arise from the interplay between cation hydration and the steric landscape of the dinuclear active sites. The hydrated radii of Li^+^, Na^+^, K^+^, and Cs^+^ are approximately 3.1, 2.7, 2.4 and 2.1 Å, respectively (Fig. S30†).^44–46^ By contrast, the salen methylene groups on the Co_2_-COP surface create a steric pore window, with minimum and maximum separations of ∼2.5 Å and ∼7.3 Å (Fig. S31†). Hydrated K^+^ and Cs^+^ cations are comparable to the pore window, so they adsorb loosely on the Co_2_-COP surface. Since K^+^ possesses a moderate hydration ability, continuously supplying fresh H_2_O to the Co sites and giving the highest HER rate. While Cs^+^ has weaker hydration capacity, delivers water less efficiently and thus shows slightly lower activity than K^+^. The hydrated Li^+^ and Na^+^ clusters are only slightly smaller than the pore window of Co_2_-COP, form a dense interfacial layer over the Co-OH* sites. Their extensive hydration shells hinder OH^−^ desorption, thereby diminishing the HER performance of Co_2_-COP.

## Conclusion

In summary, we have successfully designed and synthesized a novel dinuclear cobalt catalyst Co_2_-COP, which incorporates well-defined Co-Robson structural units with a Co–Co distance of 2.7 Å. This design effectively facilitates both water dissociation and OH* intermediate removal, showcasing the power of direct and indirect DMSC effect. Co_2_-COP exhibits outstanding electrocatalytic performance for the alkaline HER, achieving a mass activity 36 times higher than its single-atom counterpart (Co-COP). Theoretical calculations and experimental data confirm that the enhanced catalytic efficiency of Co_2_-COP stems from the favorable adsorption energies for both water and hydrogen, as well as the synergistic electronic interactions between the two Co atoms. Furthermore, the alkali-metal-cation-dependent effect, especially with K^+^, plays a crucial role in optimizing the HER process by stabilizing the OH* intermediate and enhancing charge transfer. These findings provide valuable insights into the combined influence of direct and indirect DMSC effects, paving the way for the design of more efficient dinuclear catalysts for energy conversion applications.

## Author contributions

D.-C. Zhong and W.-J. Shi conceived the idea. X.-M. Hu performed the main experiments. X.-M. Hu analyzed the data. W.-J. Shi and W.-X. Tao performed DFT calculations. J.-H. Mei and Y.-C. Wang performed the FT-IR measurements. W.-J. Shi and X.-M. Hu wrote the manuscript, D.-C. Zhong and T.-B. Lu revised the paper. All authors discussed the results and commented on the manuscript.

## Conflicts of interest

There are no conflicts to declare.

## Supplementary Material

SC-016-D5SC02073E-s001

## Data Availability

The data supporting this article have been included as part of the ESI.†
