# Supplementary material for: A salen-based dinuclear cobalt(ii) polymer with direct and indirect synergy for electrocatalytic hydrogen evolution
Source: Chem Sci. 2025 May 2;16(23):10364–71. doi: 10.1039/d5sc02073e (PMC12060030; doi:10.1039/d5sc02073e)
Supplement: SC-016-D5SC02073E-s001 [file SC-016-D5SC02073E-s001.pdf]

Supplementary Information (SI) for Chemical Science.
This journal is © The Royal Society of Chemistry 2025

Supporting Information

A Salen-based Dinuclear Cobalt(II) Polymer with Direct and Indirect Dinuclear Metal Synergistic Effect for Electrocatalytic Hydrogen Evolution

Xiao-Mei Hu,[†] Wen-Jie Shi,[†,*] Jian-Hua Mei, Yu-Chen Wang, Wei-Xue Tao, Di-Chang Zhong,* Tong-Bu Lu,*

Institute for New Energy Materials & Low Carbon Technologies, School of Material Science & Engineering, School of Chemistry & Chemical Engineering, Tianjin University of Technology, Tianjin 300384, China

†These authors contributed equally to this work.

1. Material characterization

All the chemicals were commercially available and used without further purification. The PXRD measurements were carried out on a SmartLab9KW diffractometer (Rigaku, Japan) with Cu Kα radiation (λ=1.54178 Å). Fourier transform infrared (FT–IR) spectra in the 400–4000 cm^{-1} region were recorded on a PerkinElmer Frontier Mid-IR FT-IR spectrometer using KBr pellets. Solid ^{13}C NMR experiments were characterized using a Bruker 400 MHz and XPS data were obtained using a Thermo Scientific spectrometer. Scanning electron microscopy (SEM) were acquired on an Environmental Scanning Electron Microscope with FEG (Quanta FEG 250, FEI, USA). Transmission electron microscopy (TEM) measurements were carried out on a FEI Tecnai G2 Spirit TWIN at an acceleration voltage of 120 kV. The atomic-resolution high-angle annular dark-field scanning transmission electron microscopy (HAADF-STEM) image was obtained by an aberration-corrected Titan Themis Cubed G2 60-300. The contents of Co species in the samples were determined by inductively coupled plasma mass spectrometer (ICP-MS, iCAP RQ, Germany).

2. Synthesis of 3,3',5,5'-Tetraformyl-4,4'-biphenyldiol (TFBO)

4,4’-biphenyldiol (3.72 g, 20 mmol) and hexamethylenetetramine (HMTA 9.31 g, 50 mmol) were mixed in a 100 mL three necked flask, trifluoroacetic acid (36 mL) was added and the solution was refluxed under Ar condition for 7 days. The mixture was poured into deionized water, and precipitated yellow product was filtered, and then recrystallized from hot dimethyl sulfoxide to obtain the crystallized TFBD with a yield of 65%. ^{1}H NMR (400 MHz, dimethyl sulfoxide (DMSO)-d_6, δ): 11.66 (s, 2H, OH), 10.34 (s, 4H, CHO), 8.43 (s, 4H, Ar-H).

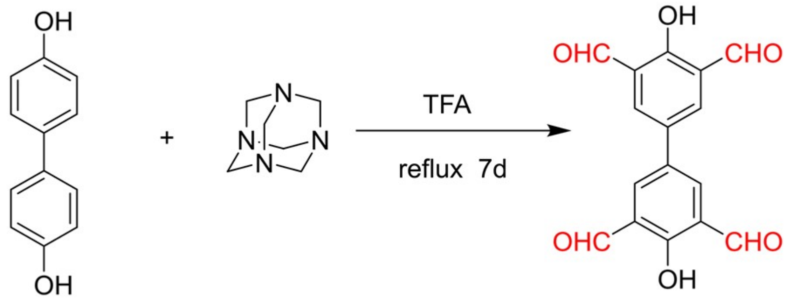

Figure S1. Synthesis of 3,3',5,5'-Tetraformyl-4,4'-biphenyldiol (TFBO).

3. Synthesis of TFBO-COP

TFBO (44.7 mg, 0.15 mmol) were dissolved with ethanol / DMSO (20mL, 10 / 1, v / v) into a 100 mL round-bottom flask and then 2,2-dimethyl-1,3-propanediamine (31 μL, 0.3 mmol) was added dropwise into the flask. The resulting mixture was stirred at 70 °C for 10 h, then, filtered the orange solid and washed with ethanol. The filtered solid was vacuum dried to obtain TFBO-COP.

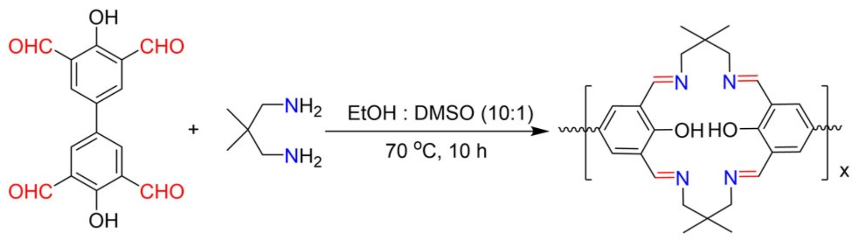

Figure S2. Synthesis of **TFBO-COP**.

4. Synthesis of DHFP-COP

4,4'-dihydroxy-3,3'-diformylbiphenyl (DHFP, 36.34 mg, 0.15 mmol) were dissolved with ethanol /DMSO (20mL, 10/1, v/v) into a 100 mL round-bottom flask and then 2,2-dimethyl-1,3-propanediamine (16 μL, 0.15 mmol) was added dropwise into the flask. The resulting mixture was stirred at 70 °C for 10 h, then, filtered the orange solid and washed with ethanol. The filtered solid was vacuum dried to obtain DHFP-COP.

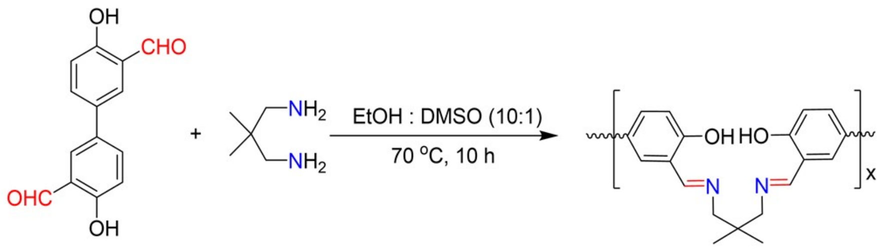

Figure S3. Synthesis of **DHFP-COP**.

5. Synthesis of Co_2-COP

TFBO-COP (20 mg) was ultrasonically dispersed in 50 mL of ethanol solution, then $Co(NO_3)_2{\cdot}6H_2O$ (291.03 mg, 1 mmol) was added. After refluxed for 10 h, the solid was separated and washed with a large amount of ethanol to remove free metal ions. The filtered solid was vacuum dried to obtain Co_2-COP.

The Co-COP were synthesis the same as that of Co_2-COP, except that TFBO-COP is replaced by DHFP-COP.

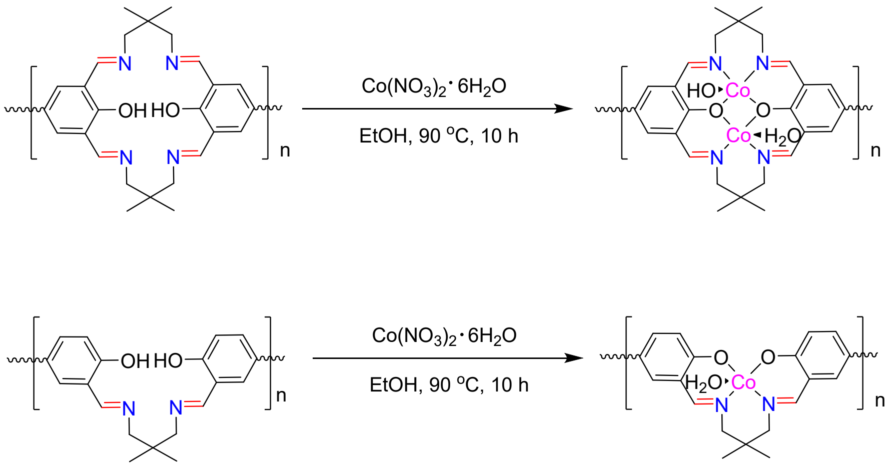

Figure S4. Synthesis of **Co_2-COP** and **Co-COP**.

6. Electrochemical measurements

Electrochemical experiments were performed on a CHI 760E electrochemical work-station using the standard three electrode setup at room temperature. The three-electrode setup comprised a working, counter and reference electrode, which were a glass carbon electrode (3 mm in diameter) coated with catalyst, a graphite rod, and Ag/AgCl (sat. KCl). The electrocatalytic performance of the electrocatalysts were tested in an N_2–saturated aqueous solution of KOH (1 mol L^{-1}). The measured potential was converted to the reversible hydrogen electrode (RHE) using the following equation at room temperature. ($E_{(RHE)}= E_{Ag/AgCl} + E_{Ag/AgCl, (sat. KCl)} + 0.059$ pH) V.

The linear sweep voltammetry (LSV) scan rate was 10 mV s^{-1}. All polarization curves were applied 85% iR compensation without additional statements. Electrochemical impedance spectroscopy (EIS) was carried out in the frequency range between 0.1 Hz to 1000 kHz and an AC voltage of 5 mV. The double layer capacitance (C_{dl}) by carrying out cyclic voltammetry at different scanning rates (20, 40, 60, 80 and 100 mV s-1) under the potential windows of 0.00–0.20 V vs. RHE. The differences in current density variation ($\Delta J=J_a–J_c$, where J_a and J_c are the anodic and cathodic current, respectively) at an overpotential of 0.10 V plotted against the scan rate and fitted to a linear regression enabled estimation of the C_{dl} for the electrocatalysts.

The catalyst ink solutions were prepared by adding 2 mg of each catalyst and 20µL of 5 wt % Nafion to a 1mL ethanol solution. The mixed suspensions were ultrasonicated for 1 h. Then, 2µL of each catalyst ink was uniformly dispersed on the polished glass carbon electrode and dried at room temperature.

7. Electrolyte Preparation for Mechanism Study

All electrochemical exploration experiments were carried out in N_2-saturated electrolyte at the scanning speed of 10 mV S^{-1} in the potential range of 0~1 V (vs RHE), and the Tafel slope was

obtained by fitting the LSV curve. Table S4 represented the electrolyte composition in different concentrations of KOH solution. Table S5 represented the electrolyte composition in the constraint K^+ ion concentration at pH=13 and pH=14 solution. Table S6 represented the electrolyte composition for studying the effect of different alkali metal (AM^+: K^+, Na^+ and Li^+) ion concentrations on HER activity Finally, we investigated the effect of different alkali metal ions on HER activity at the same concentration.

8. Infrared measurement of alkali metal cation adsorption

The **Co_2-COP** (10 mg) and **Co-COP** (10 mg) dispersed in 10 mL of methyl$_4N^+$ solution, respectively. After soaking for 24 hours, wash with water three times and dry overnight. Grind methyl$_4N^+$ solution (10 μL) and KBr (250 mg) for infrared measurement. The methyl$_4N^+$ cation show an Infrared vibration mode around 1488 cm^{-1}. Similarly, **Co_2-COP** (1 mg) and **Co-COP** (1 mg) that soaked in methyl$_4N^+$ solution were thoroughly ground with KBr (250 mg) before testing, respectively. Then, after soaking in 10 mL of methyl$_4N^+$ solution for 24 h, the **Co_2-COP** (10 mg) and **Co-COP** (10 mg) were dispersed in 1 M AMOH (AM: K^+ and Li^+, 10 mL), respectively. Finally, Centrifuge and wash with water for three times. Grind with KBr (250 mg) for infrared measurement. There is a clear exchange reaction of the form

$$\text{methyl}_4\text{N}_{ads} + \text{AM}^+_{aq} + \delta e^- \rightarrow \text{methyl}_4\text{N}^+_{aq} + \text{AM}_{ads}$$

where AM^+_{aq} and AM_{ads} represent an alkali metal cation in solution and in the adsorbed state, respectively.

9. Density Functional Theory

The density functional theory calculations were performed using the hybrid B3LYP-D3 functional (with the Grimme's D3 dispersion correction)[1-3] as implemented in the Gaussian16 program.[4] The self-consistent reaction field (SCRF) theory was used. All geometries were optimized with no constraint of freedom by using the SDD [5] pseudopotential for Co, 6-31G (d) basis set for the C, N, O, and H elements. The harmonic frequencies were then computed analytically at the same level of theory as the geometry optimizations to characterize the nature of all stationary points as minima state and to gain the Gibbs free energy correction In order to obtain more accurate relative energies, single point calculations using the def2tzvp basis set were performed based on the B3LYP-D3/mix basis set optimized molecular geometries. The 3D structures of molecules were generated using CYL View.[6]

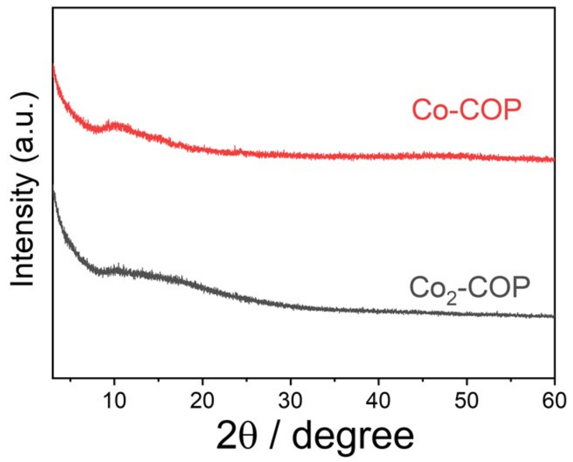

Figure S5. XRD patterns of the **Co_2-COP** and **Co-COP**.

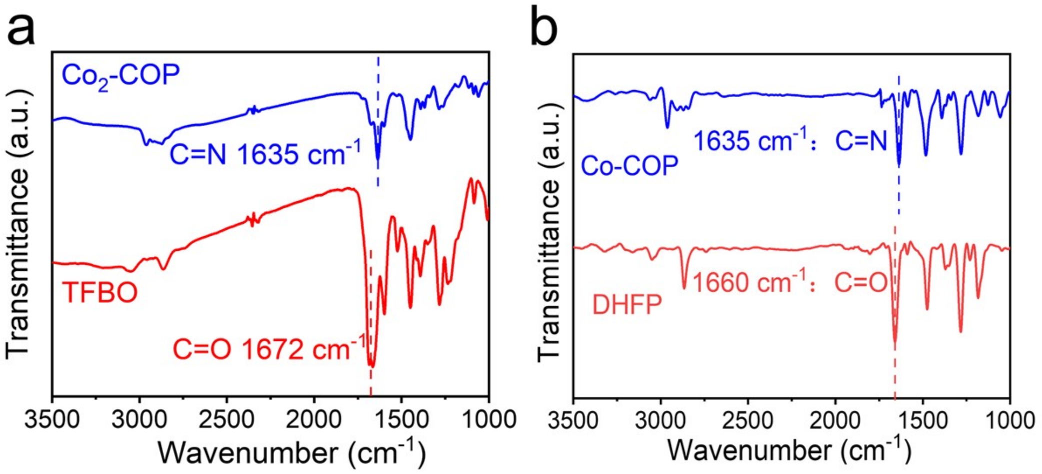

Figure S6. FT-IR spectra of (a) **Co_2-COP** and (b)**Co-COP**.

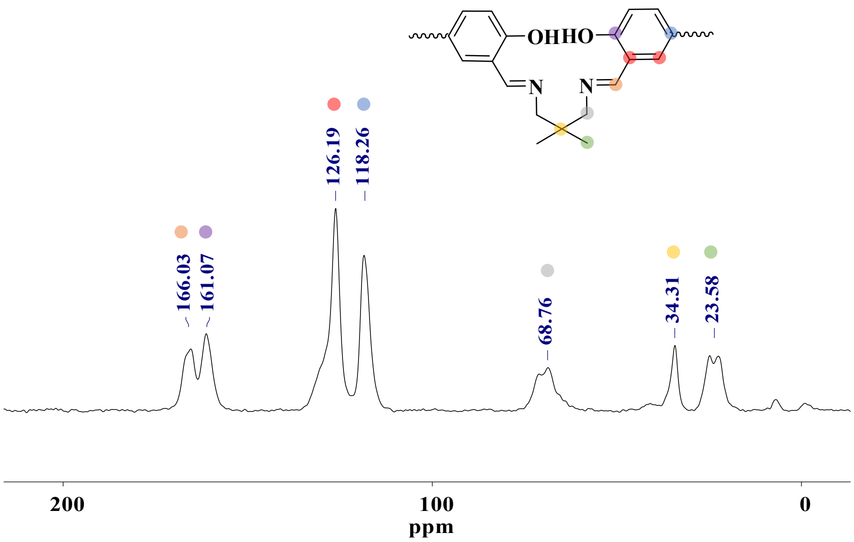

Figure S7. ^{13}C solid-state NMR spectra of **DHFP-COP**.

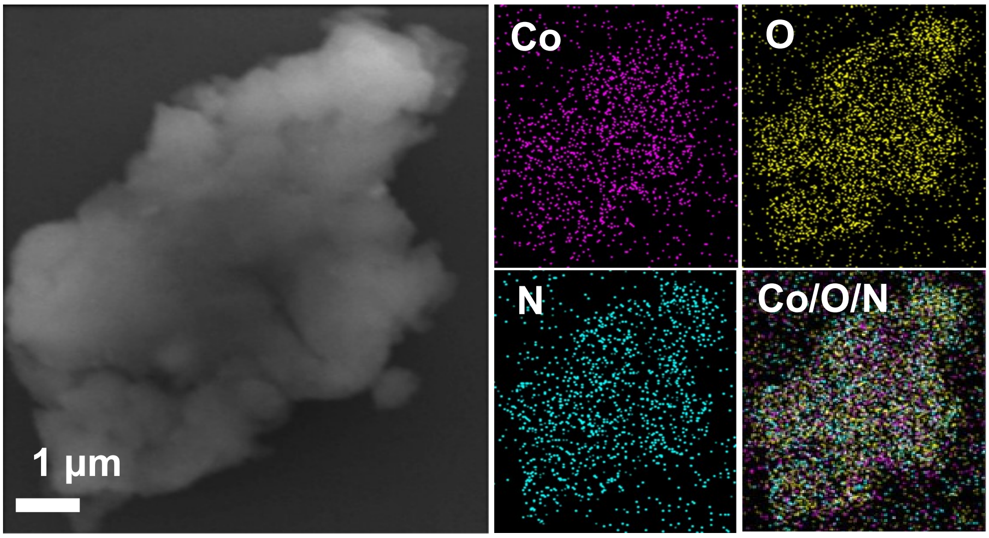

Figure S8. SEM image and corresponding elemental mapping of **Co_2-COP**.

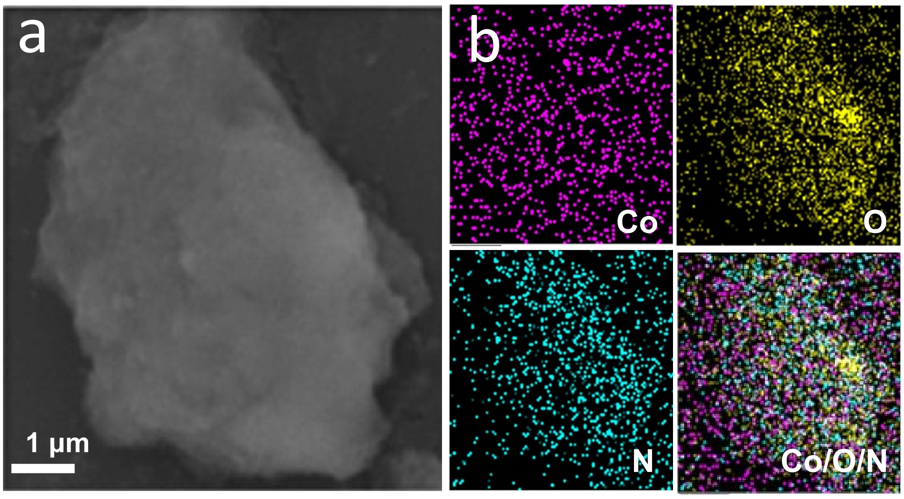

Figure S9. (a) SEM image and (b) corresponding elemental mapping of **Co-COP**.

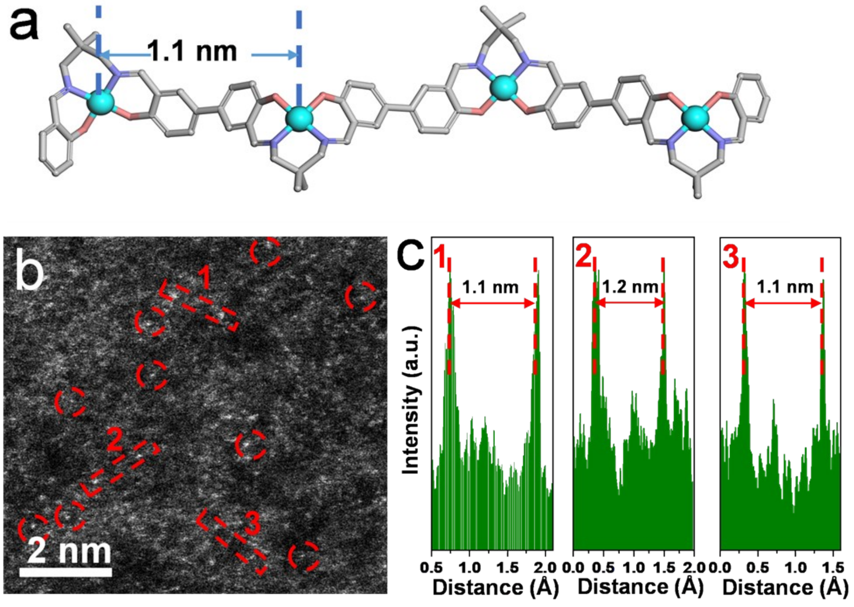

Figure S10. (a) Structural model of **Co-COP**, (b) HAADF-STEM image and (c) corresponding distances between Co in **Co-COP**.

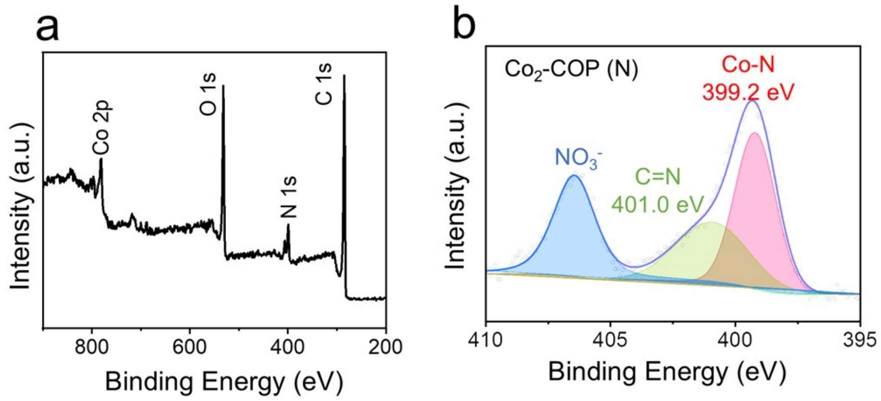

Figure S11. The XPS of **Co$_2$-COP** after HER tests. (a) Full XPS survey, (b) Co 2p, (c) O 1s, (d) N 1s.

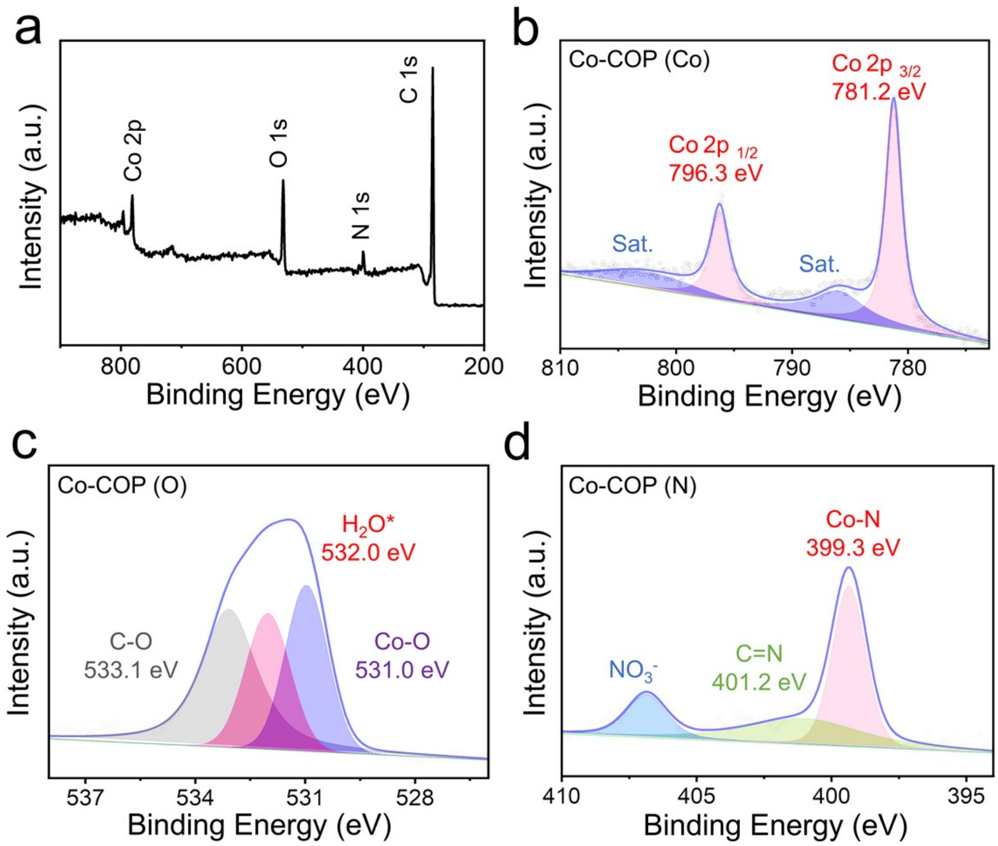

Figure S12. XPS spectra of **Co-COP** (a) Full XPS survey, (b) Co 2p, (c) O 1s, (d) N 1s.

Table S1. The contents of Co in **Co_2-COP** and **Co-COP** determined by ICP-MS.

Samples	ICP-MS (wt %)	Standard Deviation
**Co_2-COP**	11.96<br>12.09<br>11.34	0.40
**Co-COP**	10.29<br>10.77<br>10.23	0.30

Table S2. C_{dl} and η values of **Co_2-COP** and **Co-COP** for HER at current density of 10 mA cm^{-2}**.**

Samples	C_{dl} (mF/cm^2)	$\eta_{10\ mA/cm2}$ (mV)
**Co_2-COP**	0.47	540
**Co-COP**	0.53	669

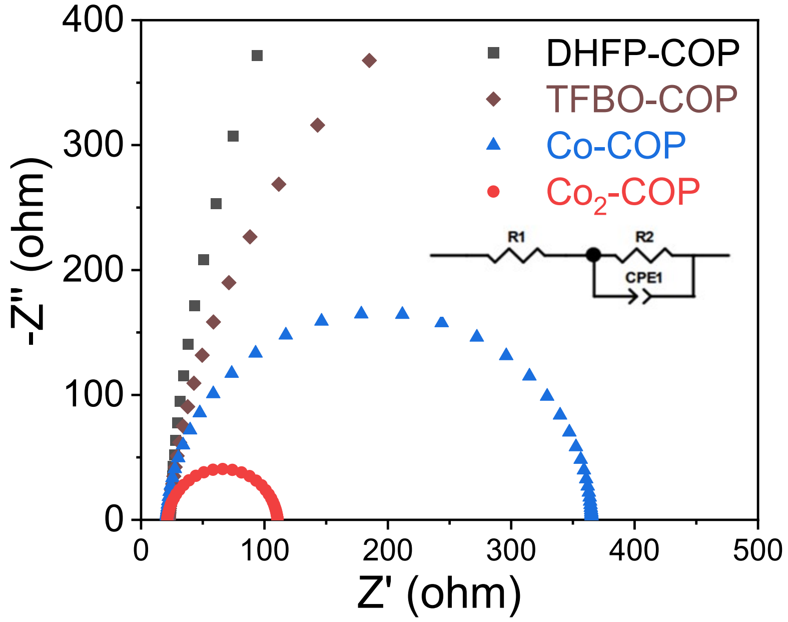

Figure S13. The equivalent circuit for EIS fitting of **Co_2-COP** (blue) and **Co-COP** (red). (R1: electrolyte resistance (Rs), R2: charge-transfer resistance (Rct), and CPE: constant-phase element).

Table S3. The R_s and R_{ct} values of **Co_2-COP** and **Co-COP**.

Samples	R_s (ohm)	R_{ct} (ohm)
**Co_2-COP**	21.29	89.38
**Co-COP**	20.77	344.5

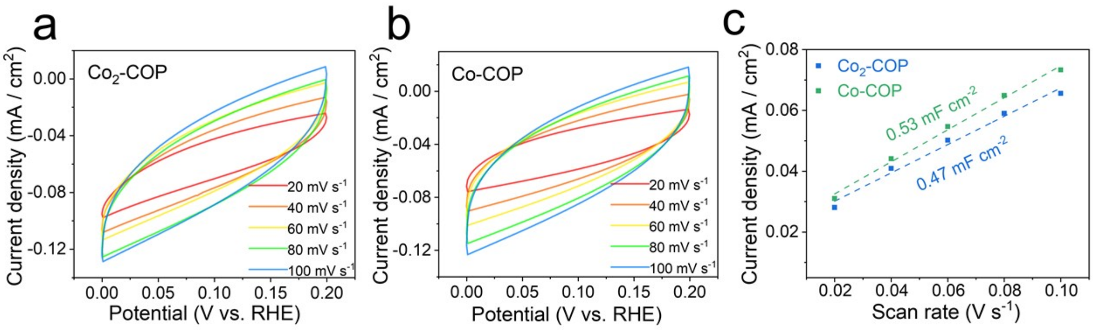

Figure S14. Cyclic voltammetry of (a) **Co_2-COP** and (b) **Co-COP** measured from 20 to 100 mV s^{-1} under alkaline medium, and (c) the corresponding plots of Δj vs. scan rates.

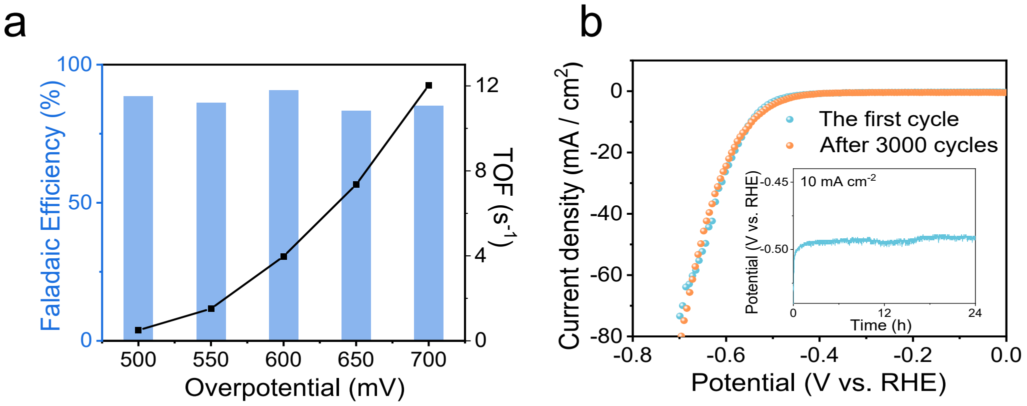

Figure S15. (a) FEs and TOF of **Co_2-COP**. (b) The polarization curves of **Co_2-COP** measured in 1 M KOH before and after 3000 CV cycles (The inset corresponds to chronoamperometric curve of **Co_2-COP** in alkaline media).

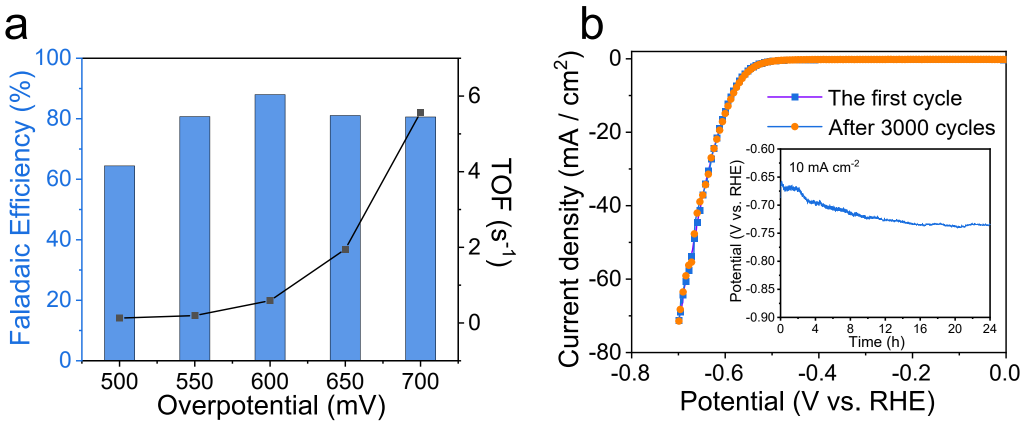

Figure S16. (a) FEs and TOF of **Co-COP**. (b) The polarization curves of **Co-COP** measured in 1 M KOH before and after 3000 CV cycles.

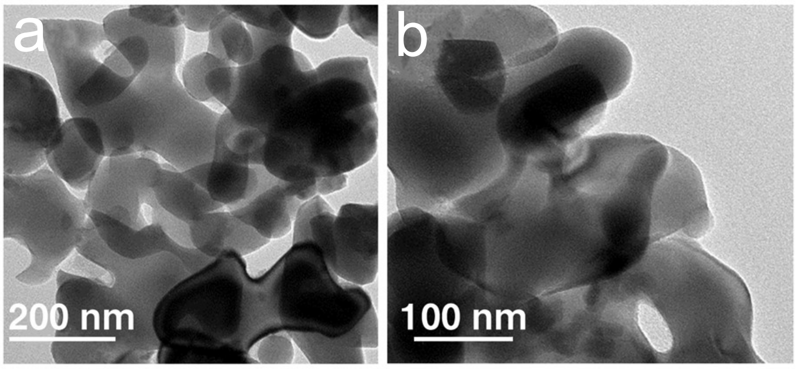

Figure S17. TEM images of $\mathbf{Co_2}$**-COP** after the HER test.

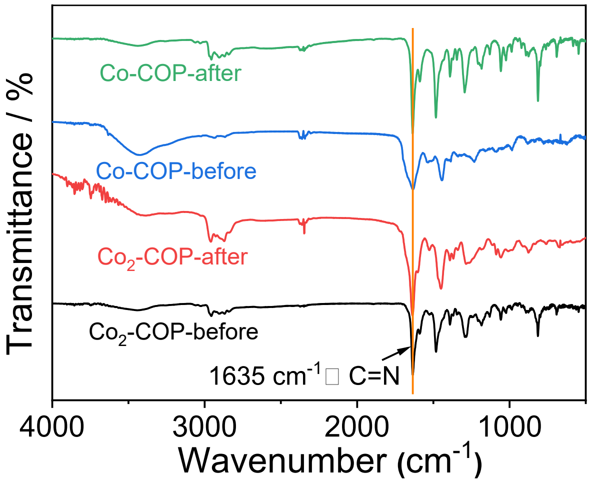

Figure S18. FT-IR image of $\mathbf{Co_2}$**-COP** before and after the HER tests.

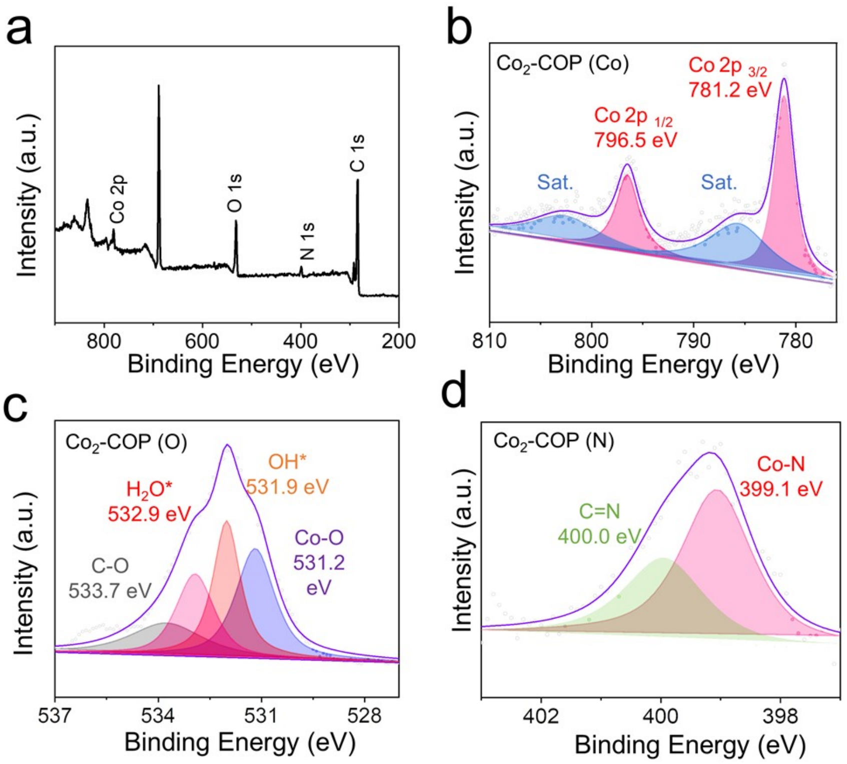

Figure S19. The XPS of **Co_2-COP** after HER tests. (a) Full XPS survey, (b) Co 2p, (c) O 1s, (d) N 1s.

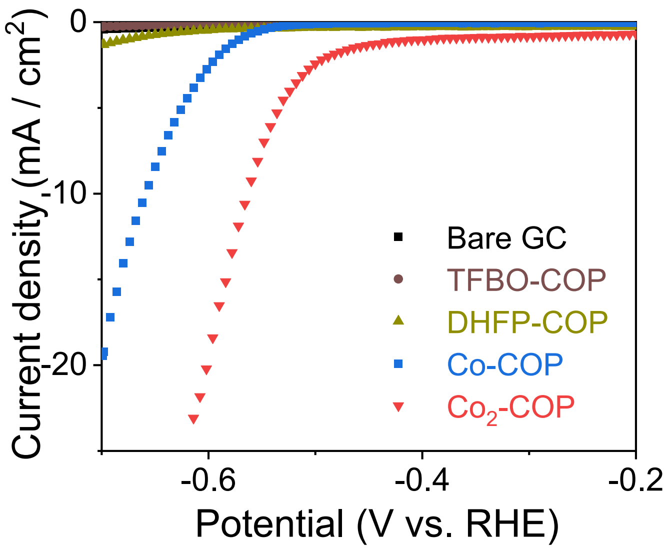

Figure S20. LSVs of **TFBO-COP**, **DHFP-COP**, **Co-COP**, **Co_2-COP** and bare GC in a KOH aqueous solution (1 mol·L^{-1}).

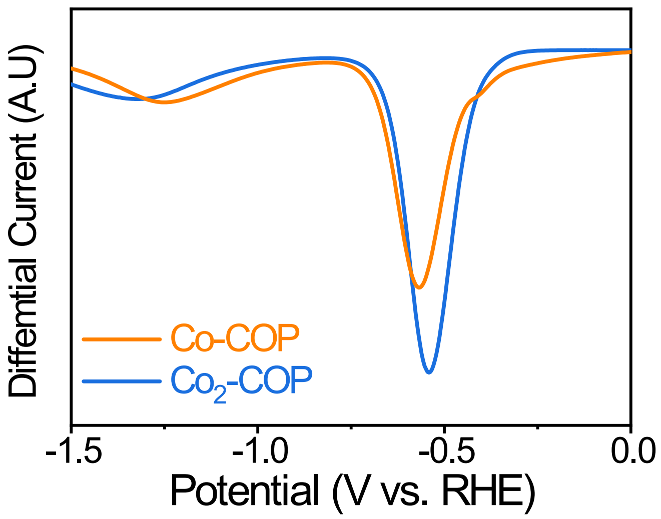

Figure S21. Differential pulse voltammetry of **Co_2-COP** and **Co-COP** in 0.1 M [TBA]PF_6 acetonitrile solution.

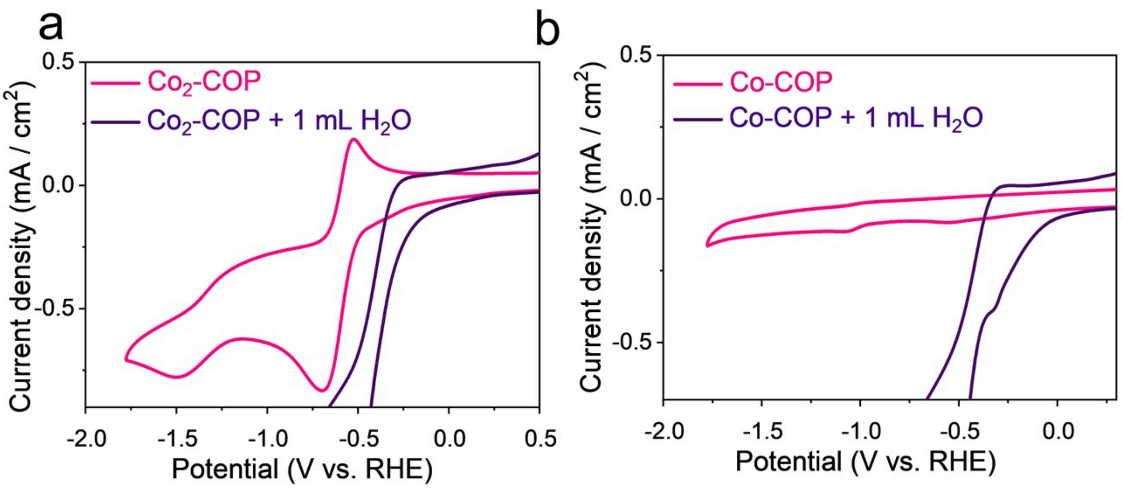

Figure S22. CV curves of **Co_2-COP** and **Co-COP** before (pink), and after addition of 1 mL H_2O (dark purple) in 0.1 M [TBA]PF_6 acetonitrile solution.

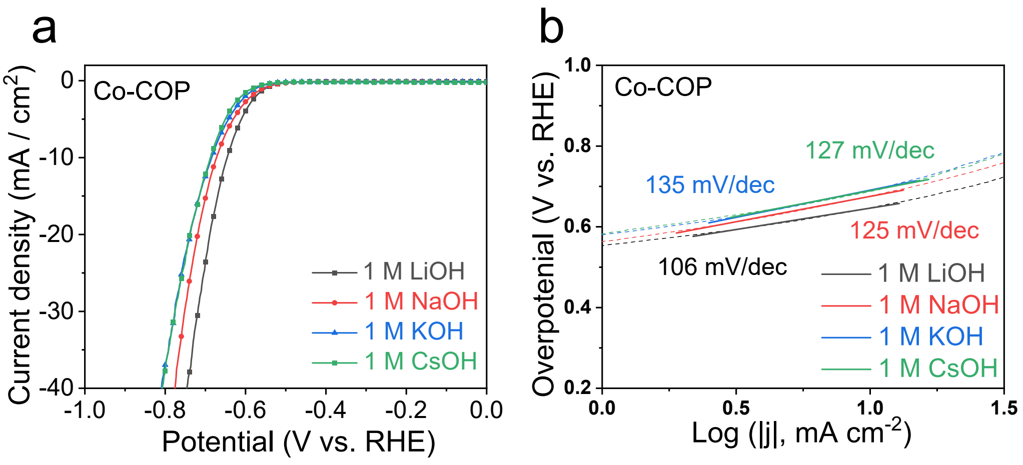

Figure S23. (a) HER polarization curves and (b) Tafel plots of **Co-COP** in 1.0 M AMOH (AM^+ = Li^+, Na^+, K^+ and Cs^+).

Table S4 The composition of the electrolyte for studying the effect of different alkali metal (AM^+: K^+, Na^+ and Li^+) ion concentrations on HER activity.

AMOH (mol L^{-1})	AMCl (mol L^{-1})	AM^+ (mol L^{-1})
0.1	0	0.1
0.1	0.3	0.4
0.1	0.9	1.0

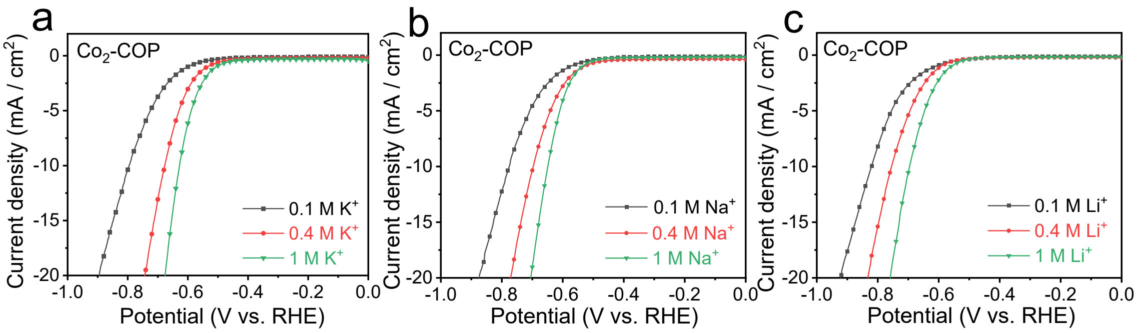

Figure S24. HER polarization curves of **Co_2-COP** in 0.1 (a), 0.4 (b) and 1 M (c) AMOH ($AM^+ = K^+$, Na^+ and Li^+).

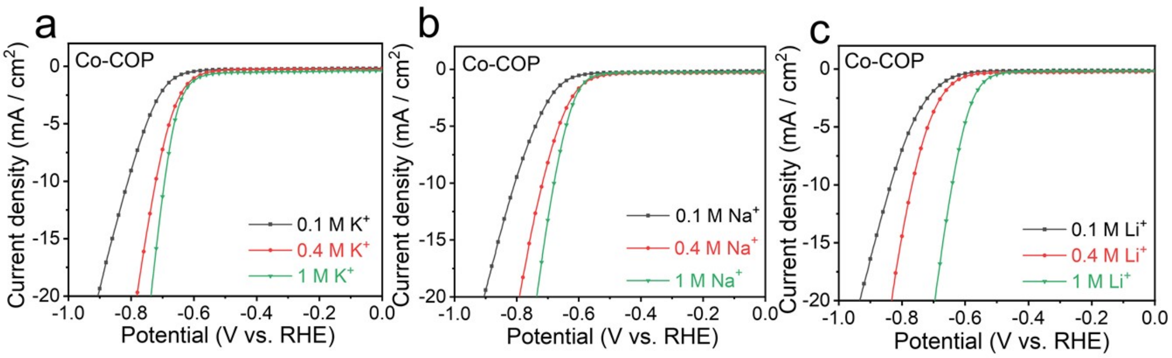

Figure S25. HER polarization curves of **Co-COP** in 0.1 (a), 0.4 (b) and 1 M (c) AMOH ($AM^+ = K^+$, Na^+ and Li^+).

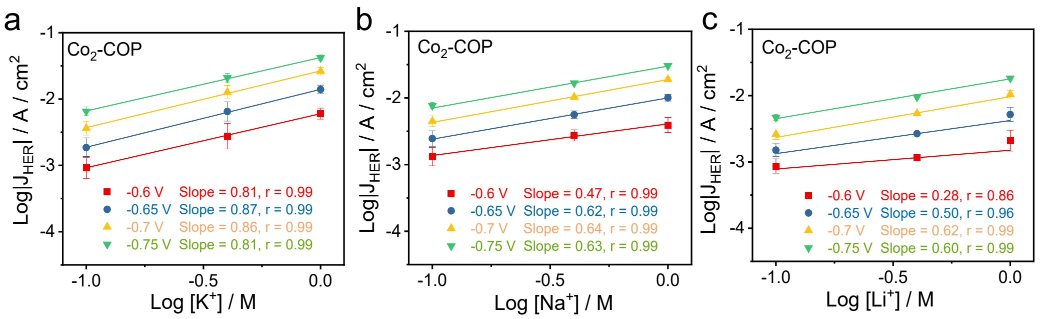

Figure S26. Reaction order plot of HER in the cation concentration at pH 13 on **Co_2-COP** ($AM^+ = K^+$ (a), Na^+ (b), and Li^+ (c)) at 50 mV potential steps (vs. RHE) plotted as a function of the logarithm of the current density on the y-axis and logarithm of the [AM^+] concentration on the x-axis. The corresponding slopes (reaction orders) are indicated next to the plots, where the slope at the bottom corresponds to the applied potential of -0.6 V (vs. RHE) and the slope at the top corresponds to the applied potential of -0.75 V (vs. RHE) in all the graphs.

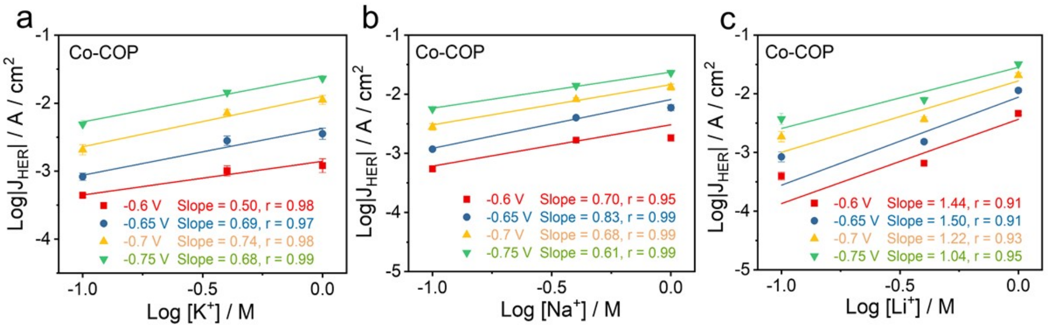

Figure S27. Reaction order plot of HER in the cation concentration at pH 13 on **Co-COP** ($AM^+ = K^+$ (a), Na^+ (b), and Li^+ (c)) at 50 mV potential steps (vs. RHE) plotted as a function of the logarithm of the current density on the y-axis and logarithm of the [AM^+] concentration on the x-axis. The corresponding slopes (reaction orders) are indicated next to the plots, where the slope at the bottom corresponds to the applied potential of -0.6 V (vs. RHE) and the slope at the top corresponds to the applied potential of -0.75 V (vs. RHE) in all the graphs.

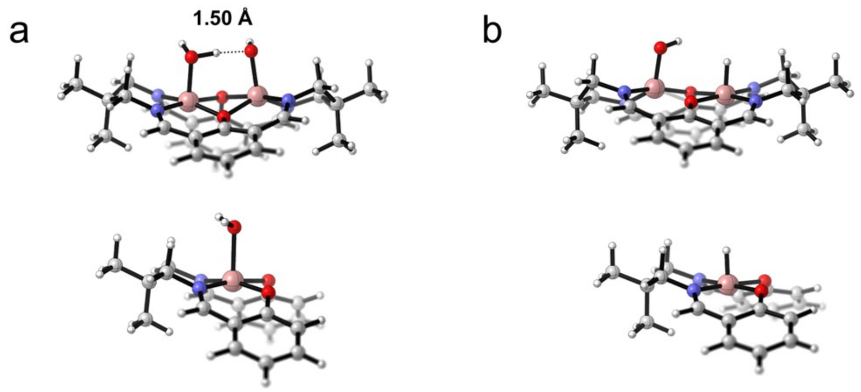

Figure S28. The structural models of **Co_2-COP** and **Co-COP** with adsorption of (a) H_2O and (b) H species.

Table S5 Electrolyte composition to study the effect of pH values on HER activity.

pH	KOH (mol L^{-1})	KCl (mol L^{-1})	K^+ (mol L^{-1})
13	0.1	0.9	1.0
14	1	/	1.0

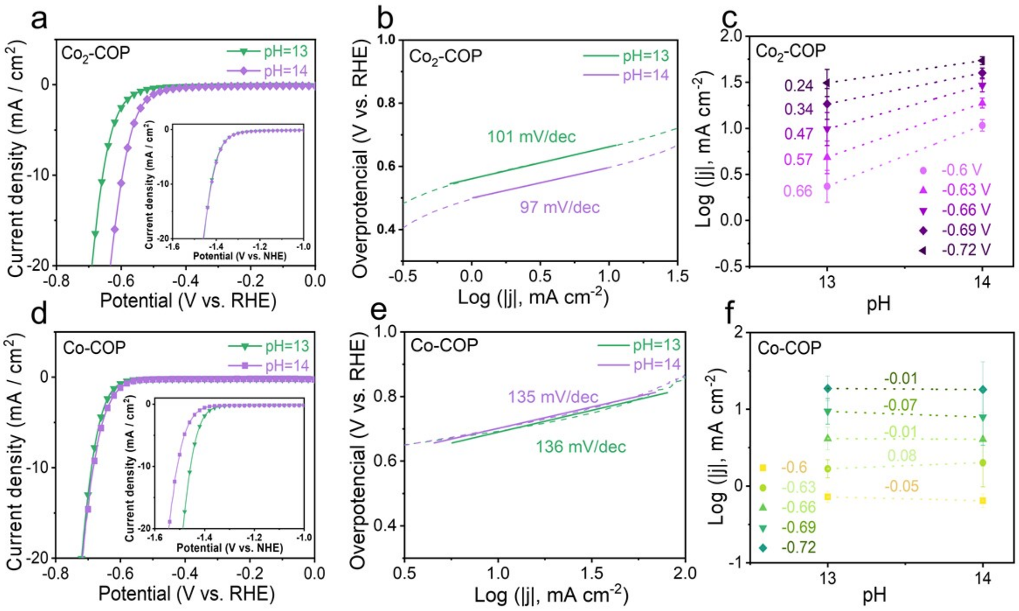

Figure S29. HER polarization curves and Tafel plots in bulk electrolyte pH for a constant concentration of cations in the bulk (1 M) on **Co_2-COP** (a, b) and **Co-COP** (d, e). All data obtained from the 0.1 M KOH + 0.9 M KCl (pH 13), 1M KOH (pH 14), in N_2 saturated environment at 10 mV/s. The potentials of NHE scale was converted from the RHE scale by the equation (E_{NHE} = E_{RHE} - 0.059pH). Reaction order of HER on **Co_2-COP** (c) and **Co-COP** (f) at 30 mV potential steps (vs. RHE) plotted as a function of the logarithm of the current density on the y-axis and bulk pH on the x-axis. The corresponding slopes (reaction orders) are indicated next to the plots, where the slope at the bottom corresponds to the applied potential of -0.60 V (vs. RHE) and the slope at the top corresponds to the applied potential of -0.72 V (vs. RHE).

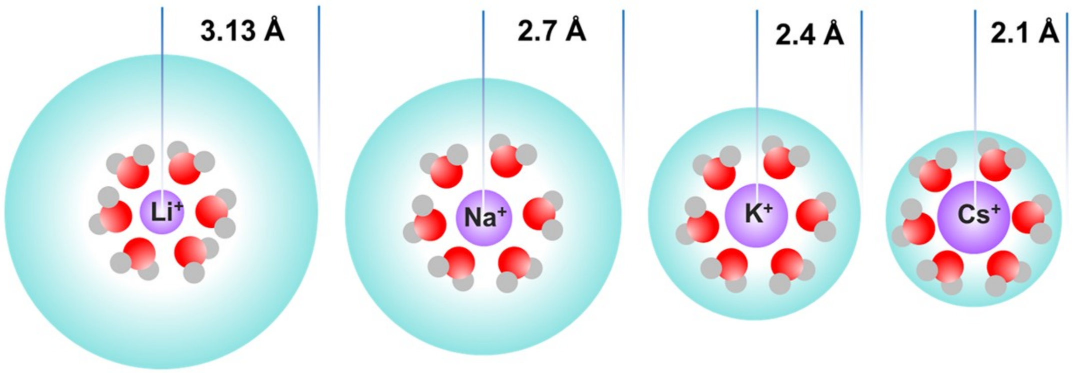

Figure S30. Scheme of the hydrated cation sizes of Li^+, Na^+, K^+ and Cs^+.

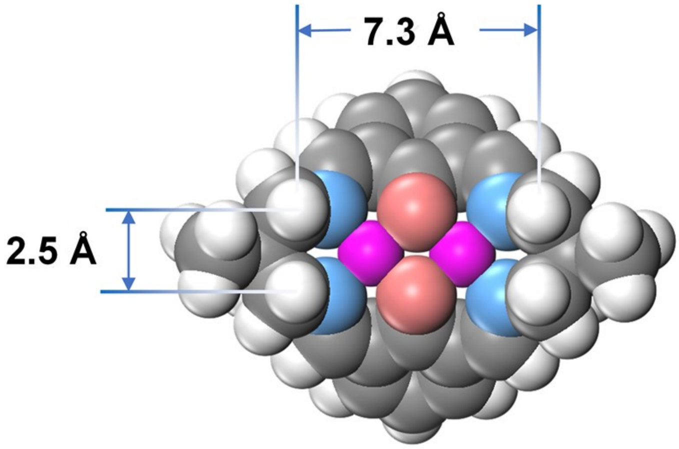

Figure 31. The local structure of **Co_2-COP**.

References

1. S. Grimme, *J. Comput. Chem.*, 2006, **27**, 1787-1799.
2. S. Grimme, J. Antony, S. Ehrlich and H. Krieg, *J. Chem. Phys.*, 2010, **132**, 154104.
3. S. Grimme, S. Ehrlich and L. Goerigk, *J. Comput. Chem.*, 2011, **32**, 1456-1465.
4. M. J. Frisch, G. W. Trucks, H. B. Schlegel, G. E. Scuseria, M. A. Robb, J. R. Cheeseman, G. Scalmani, V. Barone, G. A. Petersson and H. Nakatsuji.*Journal*, 2016.
5. D. Andrae, U. Häußermann, M. Dolg, H. Stoll and H. Preuß, *Theoretica chimica acta*, 1990, **77**, 123-141.
6. C. Y. Legault, CYLview, http://www.cylview.org.).
